# The effects of pH and NaCl concentration on the structure of β‐casein from buffalo milk

**DOI:** 10.1002/fsn3.2157

**Published:** 2021-03-09

**Authors:** Kong Yang Wu, Tong Xiang Yang, Quan Yang Li

**Affiliations:** ^1^ College of Life Science Luoyang Normal University Luoyang China; ^2^ College of Food and Bioengineering Henan University of Science and Technology Luoyang China; ^3^ College of Light Industry and Food Engineering Guangxi University Nanning China

**Keywords:** buffalo milk, circular dichroism, fluorescence spectrometry, β‐casein

## Abstract

In the present study, we aimed to investigate the effects of pH and sodium chloride (NaCl) concentration on the structure of β‐casein (β‐CN) purified from buffalo milk using circular dichroism (CD), intrinsic tryptophan, and anilino‐8‐naphthalene sulfonate (ANS) fluorescence spectroscopy. We found that NaCl concentration played a critical role in the stability of the secondary structure of β‐CN. The CD negative peak had a redshift as the NaCl concentration was increased and accompanied by a decrease of β‐sheet content and an increase of α‐helix content. ANS fluorescence spectroscopy also indicated that higher NaCl concentration and lower pH significantly affected the tertiary structure of β‐CN. Dynamic light scattering (DLS) results showed that the particle size of buffalo β‐CN had a blueshift, and then a redshift within the pH range of 5.0–7.5, and it showed a redshift when the NaCl concentration was increased.

## Graphical abstract

This study aimed to investigate the effects of pH and NaCl concentration on purified β‐casein structure from buffalo milk using circular dichroism (CD), intrinsic tryptophan and ANS fluorescence spectroscopic. It was found that NaCl concentration was a critical factor in affecting stability of the secondary structure of β‐casein.

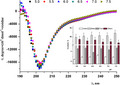

## 1 INTRODUCTION

Caseins (CNs) are the major proteins in mammalian milk, and they consist of four different protein species: α_s1_‐CN, α_s2_‐CN, β‐CN, and κ‐CN, which exist in an approximate ratio of 4:1:4:1 (w/w) in milk (Buitenhuis et al., 2016). β‐CN is one of the most abundant CNs in milk and has several unique properties, which are mostly different from other CNs (Farrell et al., 2001; Livney et al., 2004). The molecular structure of bovine β‐CN has been described previously (Livney et al., 2004; Raynes et al., 2015), and β‐CN is also considered to be a “rheomorphic protein” and molecular chaperone (Holt & Sawyer, 1993; Kehoe & Foegeding, 2011). Besides, the well‐defined amphiphilic structure of β‐CN confers excellent emulsification properties in a formulated food emulsion system, and β‐CN acts as a surfactant molecule, which can also serve as nano‐vehicles for oral drug delivery (Gangnard et al., 2007; Turovsky et al., 2015).

Many studies have demonstrated that β‐CN and its aggregated form are strongly affected by β‐CN concentration, temperature, and calcium content (Dauphas et al., 2008; Li et al., 2019; Moitzi et al., 2008; Zhang et al., 2018). Recently, studies have shown that pH affects the stability and structure of single CN microparticles (Schulte et al., 2020), and the aggregate size of β‐CN is increased with both the protein and sodium chloride (NaCl) concentrations (Jephthah, 2015). Generally, pH is an important factor determining protein structure and function. It is possible to weaken or enhance the intermolecular electrostatic interactions and hydrophobic interactions by altering the pH and NaCl concentration of the solution. Chakraborty and Basak (2007) have reported that the CNs predominantly exist in random coil conformation at neutral and alkaline pH. Ren et al. have also indicated that pH can induce changes in secondary structures of β‐CN from Chinese human milk (Ren et al., 2015). Besides, Mao et al. (2012) have studied the hydrophobic interactions of 80% milk protein concentrate (MPC) with the addition of NaCl during the diafiltration process. They have found that the addition of NaCl during the diafiltration process can modify the strength of hydrophobic interactions and sulfhydryl–disulfide interchange reactions.

Although a great deal of effort has been made in this area, most studies focus on the effects of the modification, pH, and salt concentration on the bovine β‐CN (Stroylova et al., 2011). Considering water buffalo β‐CN variants present several amino acid substitutions in bovine β‐CN (Ferranti et al., 1998), little information is available to support the effects of pH and NaCl concentration on the structures of the purified β‐CN from buffalo milk. Besides, studies barely report the changes in the native β‐CN purified from buffalo milk according to the milk‐making process. The effects of pH and NaCl concentration on the structures of buffalo milk appear to vary greatly, suggesting that the influences of pH and NaCl concentration on the β‐CN structure are complex. Therefore, in the present study, we purified native β‐CN from buffalo milk and explored the effects of pH and NaCl concentration on the β‐CN structure.

## 2 MATERIALS AND METHODS

### 2.1 Materials

Buffalo milk (7.0% fat) was purchased from the Wutang Farm. Standards of α‐CN and β‐CN were obtained from Sigma. Molecular weight marker of 10–200 kDa was supplied by Fermentas. Other chemicals of analytical grade were provided by Guangzhou Chemical Co., Ltd.

### 2.2 Purification of β‐CN

Native β‐CN was purified according to a previously described method (Neyestani et al., 2003) with minor modifications. After fresh buffalo milk was filtrated with gauze, it was defatted by centrifugation (5,860 *g*, 30 min, 4°C), and CN was precipitated and separated from buffalo milk using acetic acid at the isoelectric point (Nayak et al., 2006). Subsequently, β‐CN was enriched by calcium chloride (Toma & Nakai, 1973). The precipitate was redissolved in 30 mM Tris–HCl buffer containing 20 mM NaCl, pH 6.8 at a concentration of 100 mg/ml, followed by centrifugation at 10,000 *g* for 10 min, and then filtration through a 0.45‐µm cartridge filter. The column (2.5 × 75 cm) was filled with DEAE‐Sepharose Fast Flow and equilibrated by 30 mM Tris–HCl buffer containing 20 mM NaCl (pH 6.8). The supernatant was loaded onto the column, and proteins were eluted using a linear gradient of 0–0.5 M NaCl in the same buffer. Fractions containing purified β‐CN were dialyzed against water three times, followed by lyophilization.

### 2.3 Sodium dodecyl sulfate–polyacrylamide gel electrophoresis (SDSPAGE)

Casein samples were subjected to SDS‐PAGE on a Bio–Rad System (Mini‐Protean 3 cell) as previously described (Laemmli, 1970). The protein samples were solubilized in a solvent system after ultracentrifugation at appropriate concentrations at 20°C. Gels were stained with Coomassie blue dye for 2 hr, followed by destaining in a solution containing 30% methanol and 10% acetic acid.

### 2.4 Circular dichroism (CD)

β‐CN was dissolved in an aqueous solution containing 10 mM sodium phosphate at various pH values (pH 5.0–7.5) or NaCl at various concentrations (0.01–0.3 M at pH 7.0), followed by centrifugation at 10,000 *g* to remove any undissolved precipitates. The final concentration of the β‐CN sample was approximately 100 μg/ml. The soluble portion was filtered through a 0.45‐μm pore filter (Millipore) and used in the far‐UV CD experiments (Qi & Onwulata, 2011). The CD spectra were recorded between 190 and 250 nm at room temperature on the MOS‐450/AF‐CD‐STP‐A (Bio‐logic) using cuvettes with a path length of 1 mm and a scan time of 4.0 s/nm. Each spectrum was signal‐averaged at least three times with a resolution of 1 nm. Spectra were smoothed with Pro‐Data control software (Applied Biophysics). Measurements were corrected by subtracting the ellipticity of sodium phosphate buffer and expressed as units of deg cm/dmol/residue (Stroylova et al., 2011). Secondary structure contents were estimated using an in‐house program implementing a genetic algorithm‐based approach. Secondary structure contents were averaged over three independent deconvolution runs.

### 2.5 Fluorescence spectroscopy

Fluorescence spectra of β‐CN were recorded using a Shimadzu RF‐5301PC spectrofluorometer (Shimadzu Corp.) in a quartz cell with a path length of 1 cm as previously described (Qi & Onwulata, 2011; Stroylova et al., 2011). The slit widths were both set at 5 nm in emission and excitation pathways. The β‐CN sample prepared as described above was placed in quartz cuvettes with a path length of 10 mm. Each sample was measured in triplicate at 20°C. Emission spectra were recorded between 300 and 420 nm with an excitation wavelength of 295 nm. The scan speed was set at 60 nm/min.

### 2.6 Anilino‐8‐naphthalene sulfonate (ANS) fluorescence spectroscopy

Anilino‐8‐naphthalene sulfonate fluorescence experiments were carried out as previously described (Stroylova et al., 2011). The β‐CN sample prepared as described above was incubated at 20°C with 10 µl of freshly prepared ANS for 15 min in the dark before the analysis. For the acquisition, the excitation was fixed at 350 nm, and the emission was set between 420 and 650 nm at 20°C using a cuvette with a path length of 10 mm. Spectra of ANS fluorescence were acquired in triplicate with a Shimadzu RF‐5301PC spectrofluorometer (Shimadzu Corp.).

### 2.7 Dynamic light scattering (DLS)

Dynamic light scattering experiments were carried out by using Zetasizer Nano‐S (Malvern Instruments Limited) as previously described (Stroylova et al., 2011). The purified β‐CN samples were dissolved in 10 mM sodium phosphate buffer at various pH values (pH 5–7.5) or containing NaCl at various concentrations (0.01–0.3 M at pH 7.0) at room temperature. The index of protein was set at 1.45, and the viscosity and refractive index of the solvent were those of water, independently of the temperature. All samples were repeated in experiments. Data were analyzed using the Malvern DTS software.

## 3 RESULTS AND DISCUSSION

### 3.1 Purification of β‐CN

**FIGURE 1:**
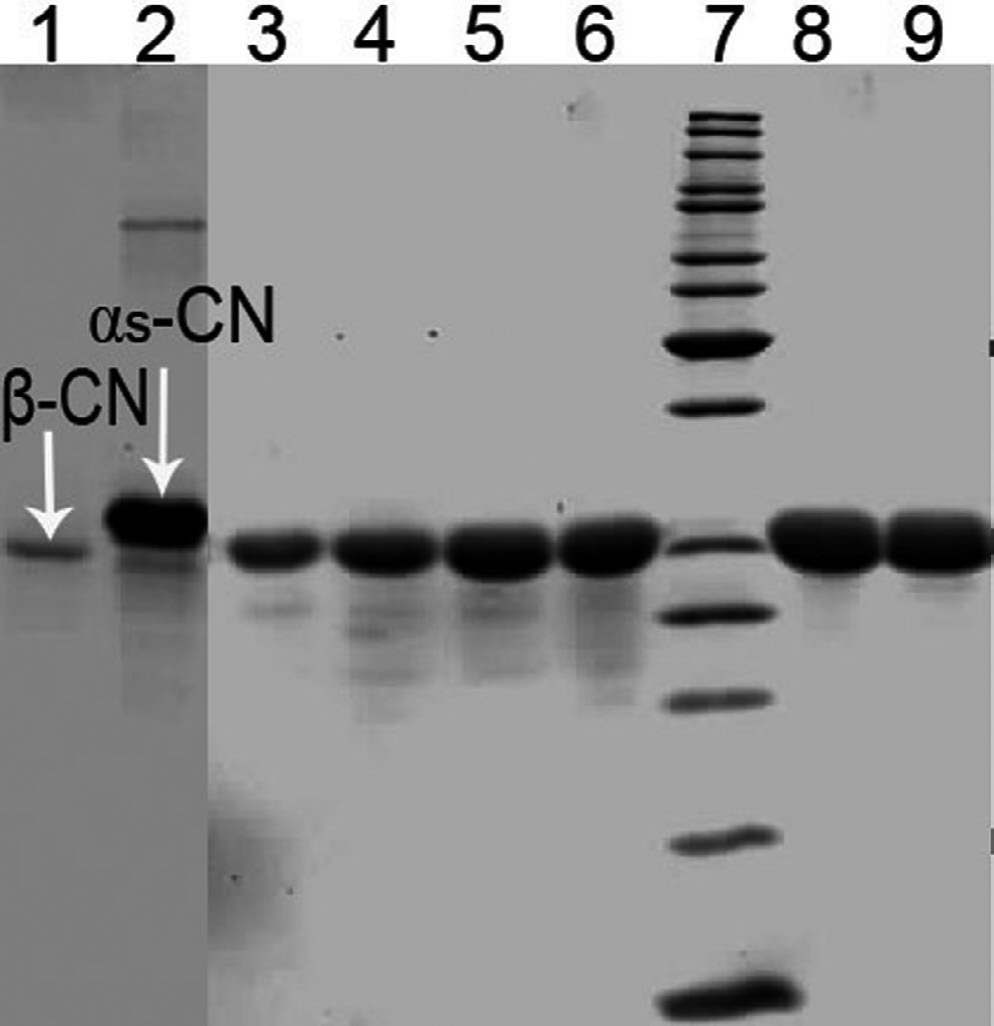
Sodium dodecyl sulfate—PAGE electrophoresis of buffalo β‐CN samples. Electrophoresis was carried out with a vertical electrophoresis system and on 12% acrylamide separating gel and 4% stacking gel with the help of standard mixtures of marker proteins. Lane 1: standard of β‐CN; Lane 2: standard of α_s_‐CN; Lane 3–6, 8, 9: purification of casein by column chromatography; Lane 7: molecular weight markers 10–200 kDa

Native buffalo β‐CN used in this study was precipitated by calcium chloride, purified by chromatography, and identified by SDS–PAGE. Figure 1 shows that the purified buffalo β‐CN (lane 8, 9) exhibited a single band and had a molecular weight of approximately 25 kDa. The molecular weight of β‐CN detected in this study was similar to that reported in other water buffalo β‐CN (Feligini et al., 2009). In general, the primary structure of β‐CN in bovine milk is composed of 209 amino acids, and its molecular mass is 23,946–24,097 Da (Livney et al., 2004).

### 3.2 Effects of pH and NaCl concentration on the secondary structure of β‐CN

**FIGURE 2:**
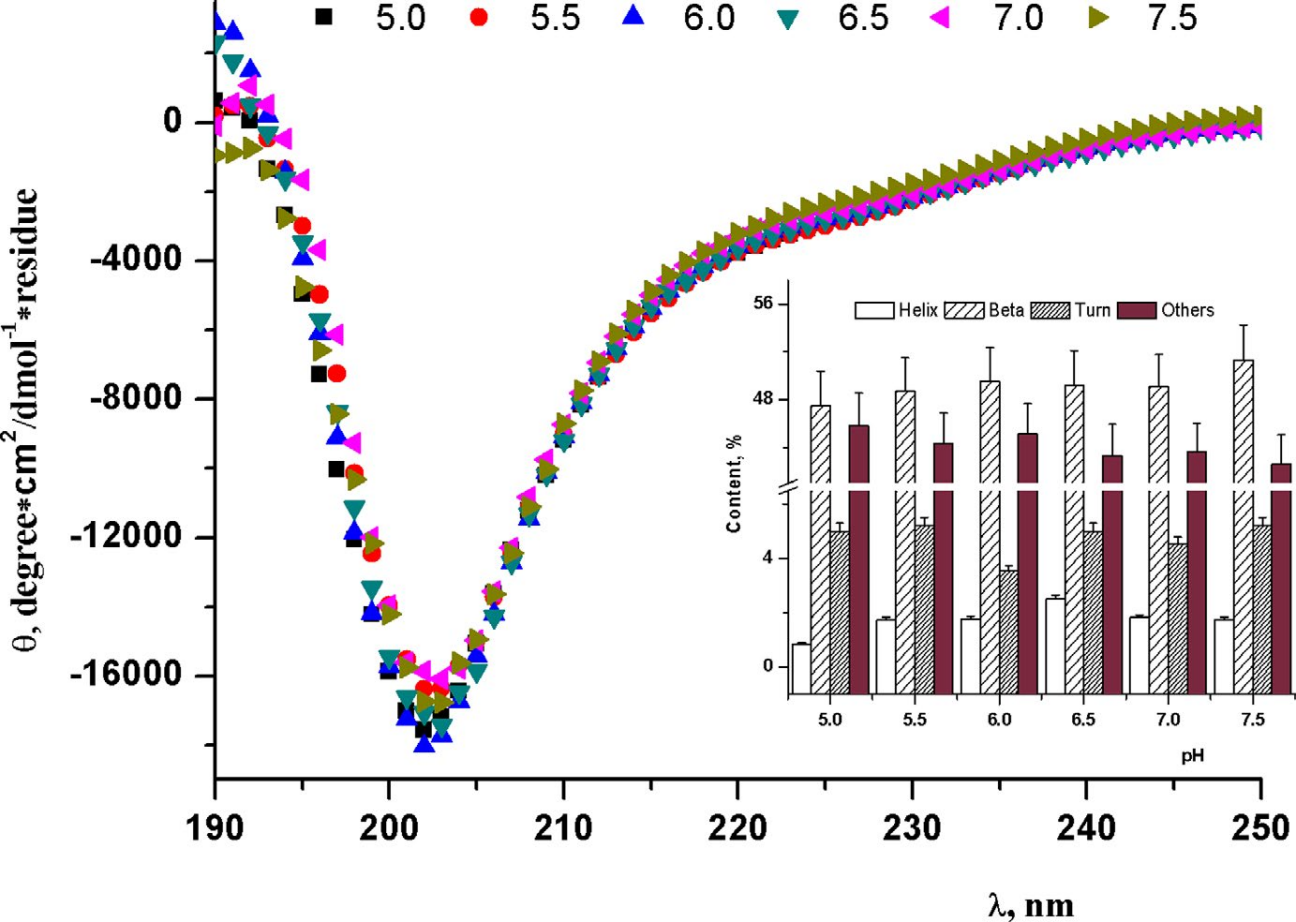
Far UV circular dichroism (CD) spectra of native buffalo β‐CN as a function of pH (5.0, 5.5, 6.0, 6.5, 7.0, and 7.5), the internal illustration is the content of α‐helix, β‐Sheet, β‐Turn changing at various pH value in 10 mM sodium phosphate buffer

Conformational changes in the secondary structure of the buffalo β‐CN were assessed according to the alterations in the CD spectra. Figure 2 represents the changes in contents of α‐helix, β‐sheet, and β‐turn at various pH values in 10 mM sodium phosphate buffer at room temperature, which were analyzed using the Chang method (Chang et al., 1978). With the decrease in pH, there was a slight redshift from 202 to 203 nm, a slight decrease in the negative value of *θ*_R‐202 nm_ at λmax, and a progressive increase in the negative ellipticity between 222 and 208 nm, while a larger change in the magnitude of *θ*_R‐208 nm_ (data were not shown) was observed. Meanwhile, the acromion between 215 and 230 nm was slightly increased after it was decreased, suggesting that the α‐helix content was increased. Moreover, the turning point (pH 6.5) emerged. This result was confirmed by the internal illustration, showing that the variable trend descended and then ascended with the decrease in pH. Conformations of α‐helix and β‐sheet were decreased from 3% to 1% and from 59% to 57%, respectively, and both reached their maximum quota at pH 6.5. Both β‐turn and random coil were increased with a decrease in pH. This finding showed that the secondary structure of buffalo β‐CN was relatively stable at pH 6.5. β‐CN consists of 3% α‐helix, 49% of β‐sheet, 5% of β‐turn, and 43% of random coil. The data were similar to the result achieved by Graham (Graham et al., 1984). Yang et al. have found that the size of CN micelles remains constant with the decrease in pH from 8.2 to 5.8, while it is sharply increased at pH ≤ 5.4 (Yang et al., 2018). This is indicative of random coil conformations replaced by ordered conformations, such as helices and sheets. In summary, pH did not seem to exert a significant effect on the secondary structure of buffalo β‐CN, although pH 6.5 could somewhat prevent the loss of the secondary structural elements of buffalo β‐CN compared with the other pH values.

**FIGURE 3:**
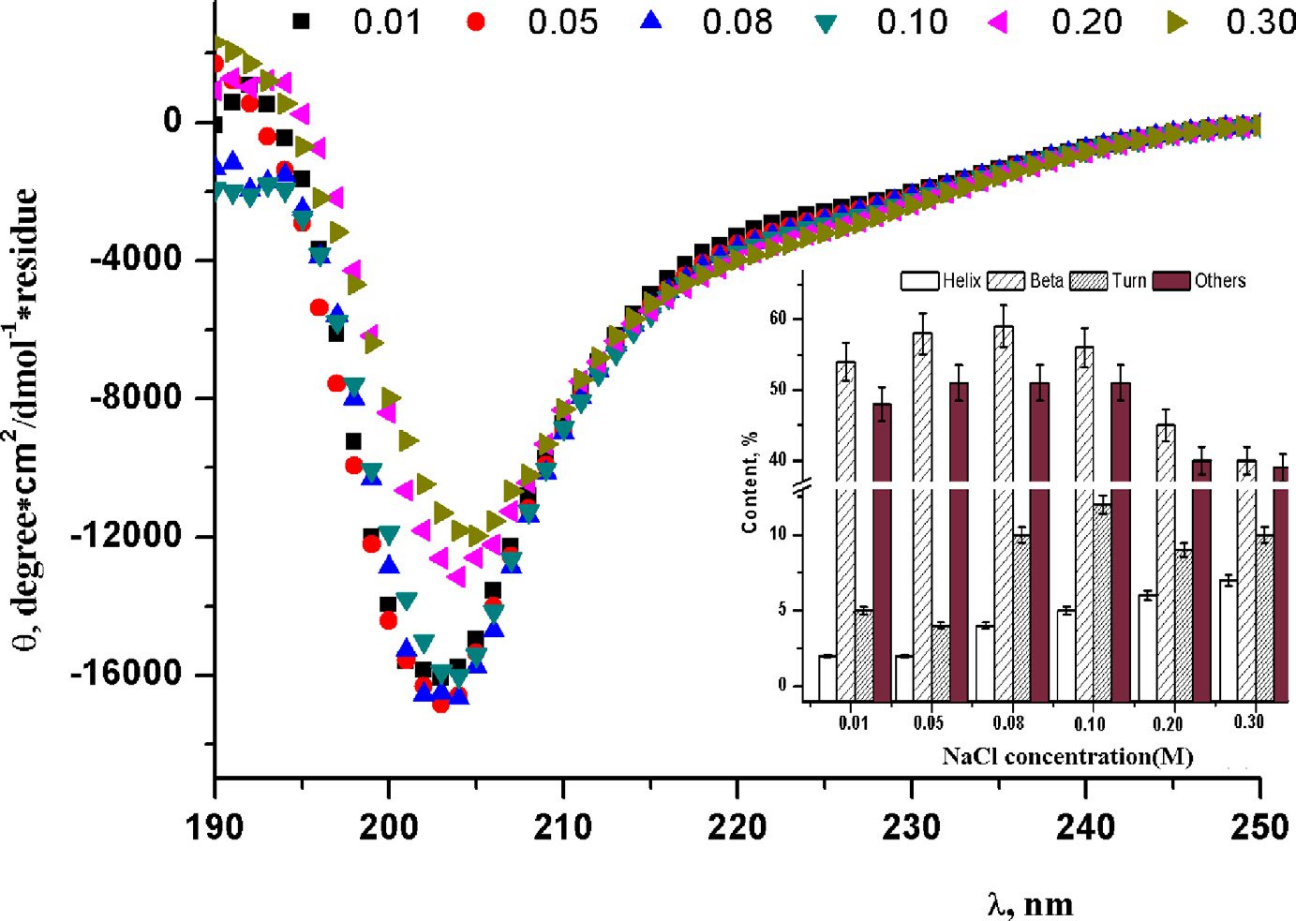
Far UV circular dichroism (CD) spectra of native buffalo β‐CN as a function of NaCl concentration (0.01, 0.05, 0.08, 0.1, 0.2, and 0.3 M), the internal illustration is the content of α‐helix, β‐Sheet, β‐Turn changing at various NaCl concentration

To explore the effect of NaCl concentration on the secondary structure of buffalo β‐CN, the native buffalo β‐CN was redissolved in the sodium phosphate buffer at various NaCl concentrations (0.01~0.3 M) at pH 7.0 (Figure 3). Figure 3 shows that the calculated secondary structural features in terms of α‐helix, fold, β‐turn, and random coil were changed with the NaCl concentration at room temperature, pH 7.0. With the increase in NaCl concentration from 10 to 300 mM, the maximum negative CD peak at 200 nm was reduced along with the simultaneous decrease in magnitude and redshift of the negative peak from 202 to 205 nm in the buffalo β‐CN, indicating a protein with an almost complete random coil structure. However, the increase in α‐helix content was noted. Meanwhile, the far‐UV CD spectra of the negative peak between the acromions of 215–230 nm were also gradually decreased, and the *θ*_R‐222 nm_ was reduced with the increase in NaCl concentration, while the *θ*_R‐208 nm_ was increased. It suggested that with the increase in NaCl concentration, the α‐helix content was increased. This result could also be observed from the internal illustration of Figure 3, showing that the α‐helix content was increased with the increase in NaCl concentration. Nevertheless, the β‐sheet content was reduced. Therefore, there was an evident effect of NaCl concentration (from 0.01 to 0.3 M) on the secondary structure of buffalo β‐CN. It also explained the irregular nature of the β‐CN secondary structure.

This study demonstrated that NaCl concentration played a critical role in the secondary structure of buffalo β‐CN. As the NaCl concentration was increased, the negative CD peak had a redshift, accompanied by a decrease in β‐sheet content and an increase in α‐helix content, suggesting that high NaCl concentration resulted in the loss of the secondary structure of buffalo β‐CN. The changes in various CD specific parameters at different pH (pH 5.0–7.5) values did not result in a significant shift, while a slight change in magnitude was observed. This could likely be attributable to the increase of intervention of hydrogen bond in unordered structure (Saxena & Wetlaufer, 1971). However, pH plays a critical role in the secondary structure of proteins at a high protein concentration and high pH value (Chakraborty & Basak, 2007). The strong‐negative CD spectrum in the region of 190–205 nm signified a disordered structural element in proteins. Currently, some studies have investigated the contents of α‐helix, β‐sheet, β‐turn, and unordered contents of bovine β‐CN, with great differences among these reports. Nevertheless, the α‐helix, β‐sheet, β‐turn, and unordered contents of buffalo β‐CN barely change with pH or NaCl concentration. Qi et al. (2005) have reported that nearly 17% α‐helix, 2.5% β‐sheet, 16% β‐turn, and 65% unordered bovine β‐CN are detected at low NaCl concentration (0.05 M) and pH 6.75. Farrell et al. (2002) have reported the β‐CN with the presence of approximately 34% β‐sheet, 29%β‐turn, 32% remainder, and 5% helix for both CN peptides. The same authors have also indicated that the contents obtained by FTIR are significantly different from those obtained by far‐UV CD. Horne (Horne, 2002) has shown that the content of α‐helix assigned ranges from 13% to 29%, and the contribution from irregular structure moves from 72% to 4%. Even with assignments from the same laboratory using different techniques, the α‐helix content in β‐CN can differ by a factor of two (9%–17% and 13%–29%, respectively). The higher β‐sheet content and lower influence were found in buffalo β‐CN under conditions of various pH values and NaCl concentrations in our current study, which could be mainly attributed to the low‐protein concentration and narrow pH range.

### 3.3 Effects of pH and NaCl concentration on the tertiary structure of buffalo β‐CN

#### 3.3.1 Effects of pH and NaCl concentration on the tertiary structure of buffalo β‐CN by intrinsic marker Trp

Fluorescence of tryptophan fluorophore depends to a great extent on its close microenvironment. Therefore, intrinsic Trp fluorescence can be used to assess the tertiary structural changes in the buffalo β‐CN as a function of pH and NaCl concentration. Native buffalo β‐CN has one Trp residue in 157, and its fluorescence can be used as a probe to study environmental changes.

**FIGURE 4:**
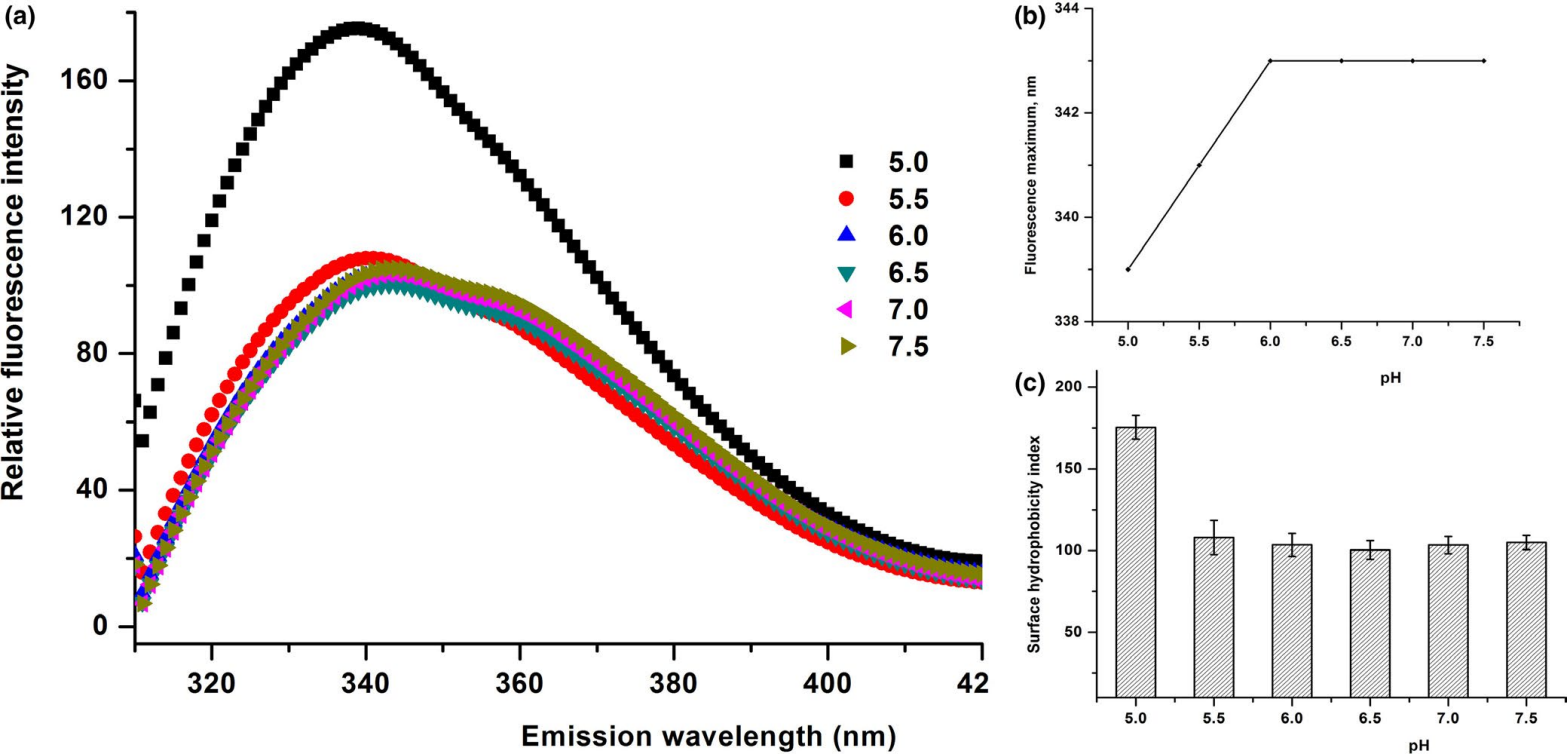
Tryptophan in trinsic fluorescence spectra of buffalo β‐CN (a), λmax (b), surface hydrophobicity index (c) as a function of pH value (5.0, 5.5, 6.0, 6.5, 7.0, and 7.5) in 10 mM sodium phosphate buffer

In the present study, we used pH and NaCl concentration as perturbation tools to assess the residual tertiary structural contacts in the β‐CN and to investigate the effects of pH and NaCl concentration on these contacts. There was no evident effect of pH on the Trp fluorescence of β‐CN in the current study, except for a pH lower than 5.0, imposing a distinct impact on the Trp fluorescence of β‐CN (Figure 4a). A gradual increase in the fluorescence intensity was observed, accompanied by a blueshift to its maximum fluorescence (from 343 to 339 nm) due to pH, especially at pH 5.0 (Figure 4b), and the surface hydrophobicity index reached 180, instantaneously (Figure 4c).

**FIGURE 5:**
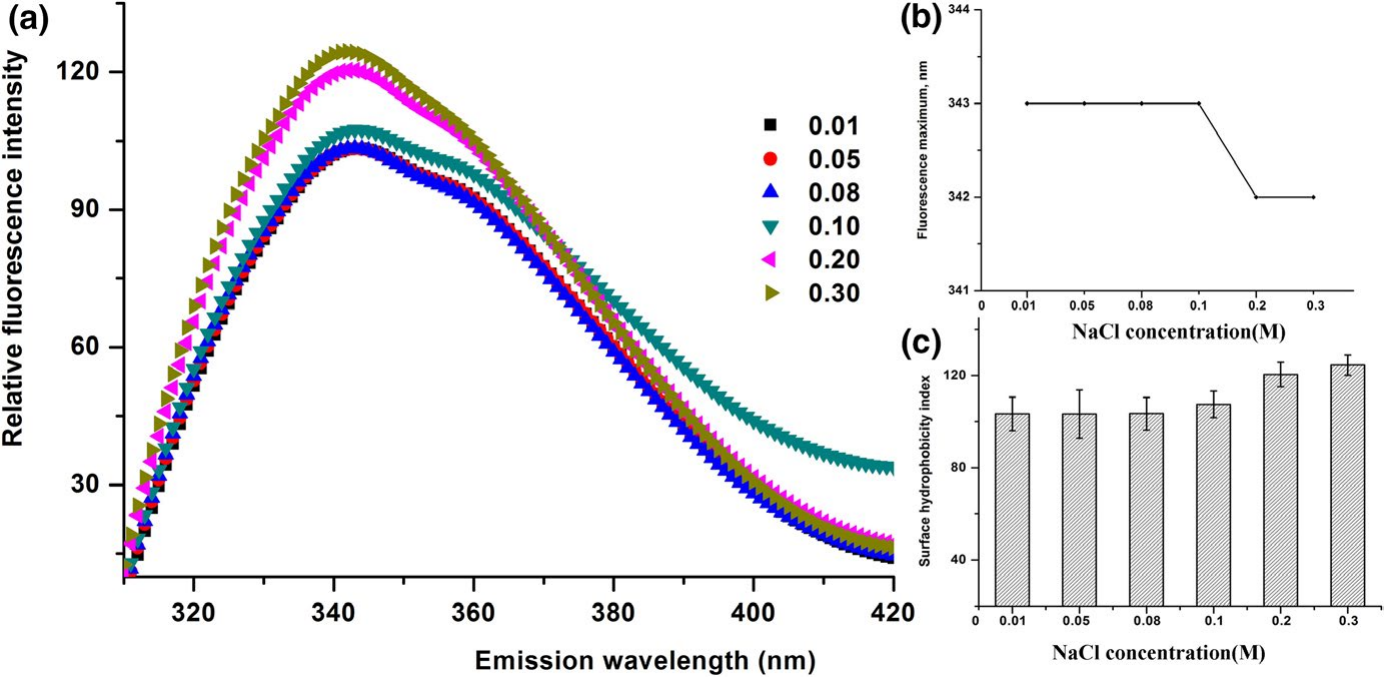
Tryptophan in trinsic fluorescence spectra of buffalo β‐CN (a), λmax (b), surface hydrophobicity index (c) as a function of NaCl concentration (0.01, 0.05, 0.08, 0.1, 0.2, and 0.3 M) at pH 7.0

Figure 5a shows that the NaCl concentration imposed a significant impact on the Trp fluorescence of β‐CN at the high NaCl concentration (≥0.2 M), coupled with slightly shorter wavelength at 342 nm (moving from 343 nm) (Figure 5b) and an increased surface hydrophobicity (Figure 5c). Moreover, pH and NaCl concentration were not disregardful influences on protein structure through electrostatic and hydrophobic interactions and a hydrogen bond. These results indicated that the low pH and high NaCl concentration caused the severe near‐complete loss of tertiary structural contacts in the Trp residues. Stroylova et al. (2011) have reported that there is a blueshift to its maximum fluorescence on the homocysteinylation of native bovine β‐CN at high temperatures. It indicates that high NaCl concentration, low pH, and high temperature can lead to the exposure of hydrophobic groups. Furthermore, the micellization and aggregation are proceeding meanwhile.

#### 3.3.2 Effects of pH and NaCl concentration on the tertiary structure of buffalo β‐CN by ANS

Binding of ANS with nonpolar regions of protein leads to a blueshift to its maximum fluorescence and rise of its fluorescence intensity (Matulis et al., 1999). The strong affinity of ANS to proteins in the “molten globule” state is caused by the lack of tertiary structure (Semisotnov et al., 1991). The tertiary structure of CN has not been well defined (Stroylova et al., 2011). In the present study, native β‐CN treated at various pH values and NaCl concentrations demonstrated an apparent increase in ANS fluorescence and a blueshift to its maximum fluorescence.

**FIGURE 6:**
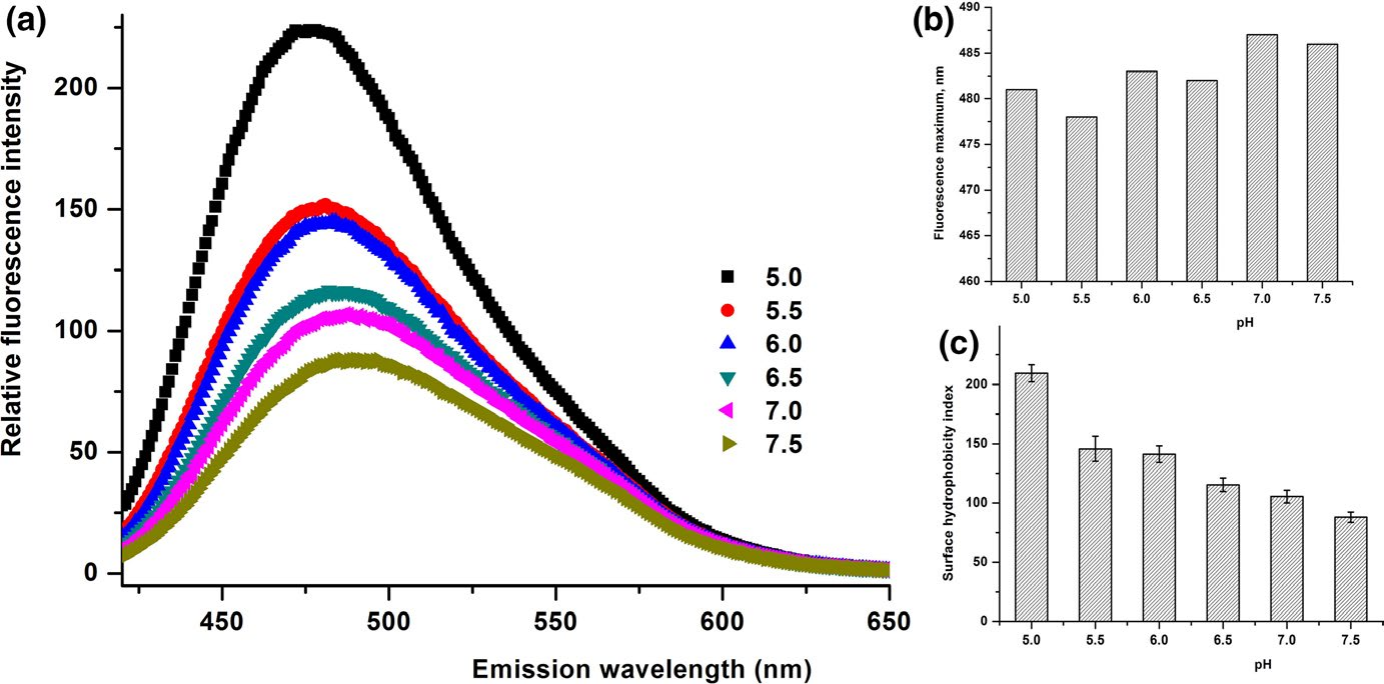
The relative fluorescence intensity of buffalo β‐CN (a), λmax (b), surface hydrophobicity index (c) treated by pH (pH 7.5, 7.0, 6.5, 6.0, 5.5, and 5.0) in 10 mM sodium phosphate buffer using 1‐anilinonaphthalene‐8sulfonic acid probe (ANS)

**FIGURE 7:**
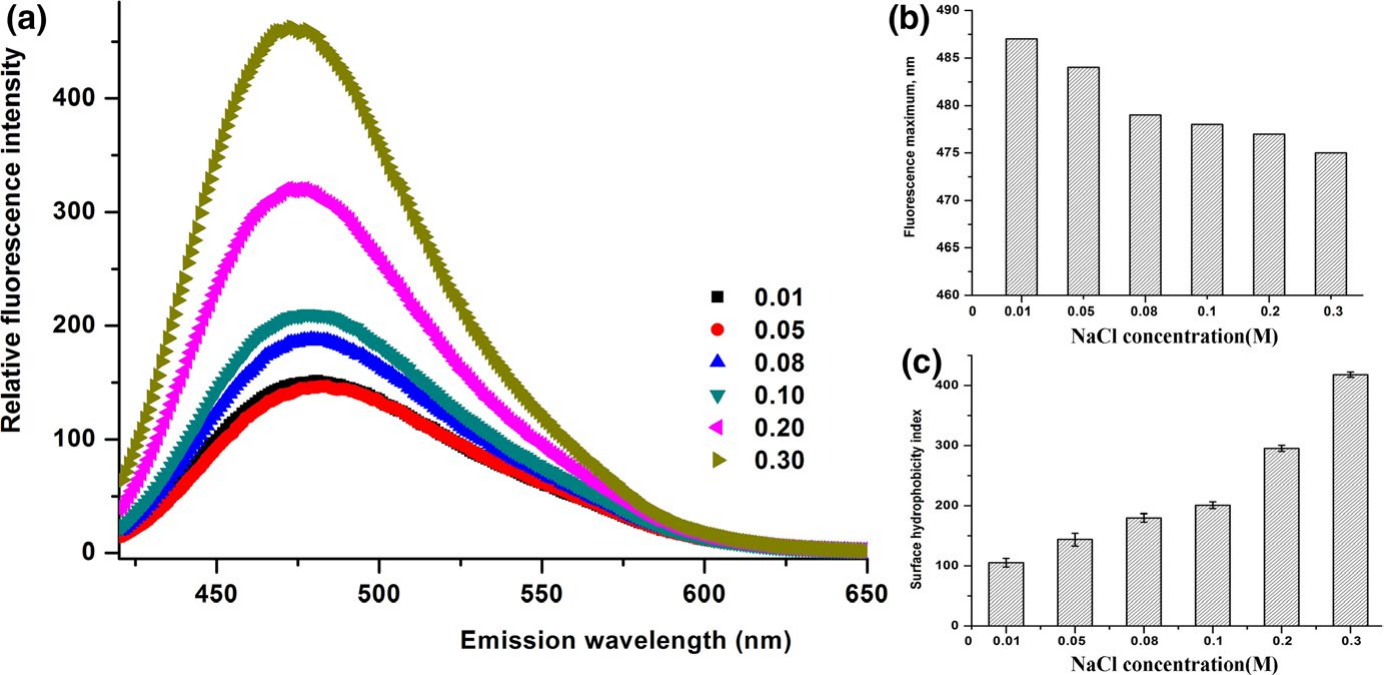
The relative fluorescence intensity of buffalo β‐CN (a), λmax (b), surface hydrophobicity index (c) treated by various NaCl concentration (0.01, 0.05, 0.08, 0.1, 0.2, and 0.3 M) at pH 7.0, in 10 mM sodium phosphate buffer using 1‐anilinonaphthalene‐8sulfonic acid probe (ANS)

Figures 6 and 7 present the relative fluorescence intensity of buffalo β‐CN treated at various pH values and NaCl concentrations, respectively. There was a visible blueshift to its maximum fluorescence (from 481 to 475 nm) (Figure 6). With the decrease in pH, the relative fluorescence intensity of buffalo β‐CN was obviously increased with a blueshift to its maximum fluorescence (from 486 to 481 nm) (Figure 6). Furthermore, the relative fluorescence intensity of buffalo β‐CN treated at a higher NaCl concentration was significantly higher than that at a lower NaCl concentration (Figure 7).

At each pH and NaCl concentration, ANS fluorescence seemed to exert a significant effect on the protein tertiary structures of the buffalo β‐CN, especially at a lower pH and a higher NaCl concentration. The increase in ANS‐hydrophobicity might be attributed to the unfolding of the buffalo β‐CN, leading to the exposure of the hydrophobic interior of the protein. The increase in surface hydrophobicity indicated that pH and NaCl concentration resulted in a rearrangement of buffalo β‐CN structure, and more hydrophobic regions in the buffalo β‐CN were exposed by a lower pH (pH 5.0) and a higher NaCl concentration (0.3 M).

These findings could be explained by the possibility that the interactions between hydrophobic “tails” blocked the interactions of β‐CN with ANS and the electrostatic repulsion between proteins (Mao et al., 2012).

### 3.4 Effects of pH and NaCl concentration on the particle size distribution of the buffalo β‐CN by DLS

**FIGURE 8:**
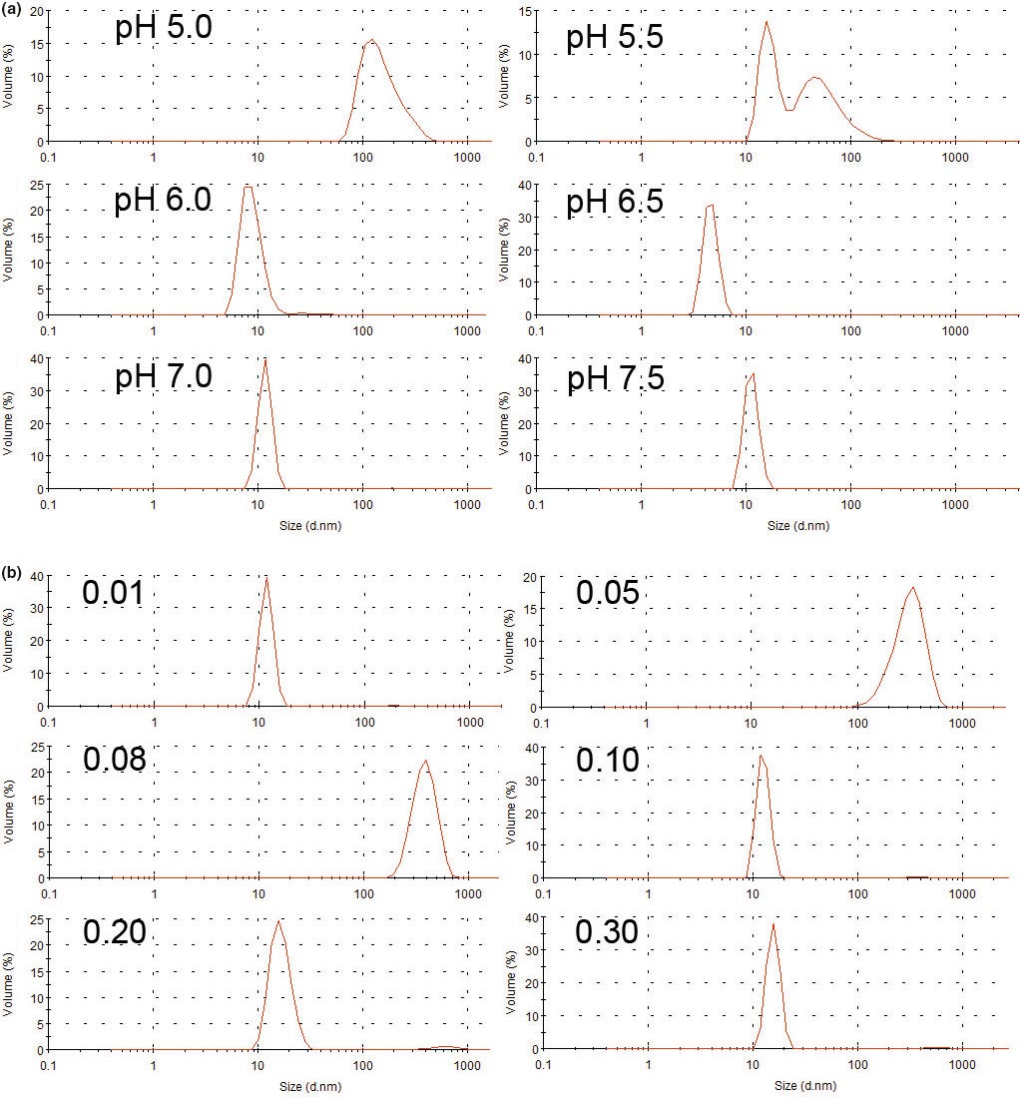
Distribution of hydrodynamic diameters of buffalo β‐CN particles treated by different pH (a) and NaCl concentration (b)

Dynamic light scattering was applied to investigate the changes in the particle size distribution of buffalo β‐CN as a function of pH (Figure 8a) or NaCl concentration (Figure 8b) at room temperature. Figure 8a shows that buffalo β‐CN was stable as a monomer at its natural pH, displaying a particle size distribution of about 10 nm. However, with the decrease in pH value, the protein particles were gradually gathered. The aggregation appeared at pH 5.5 and visibly formed as micelles with a particle size of 100 nm at pH 5.0. Moreover, its particle size distribution also changed with NaCl concentration. Buffalo β‐CN presented as a monomer in 10 mM NaCl, displaying a particle size distribution of 10 nm. With the increase in NaCl concentration (from 0.05 to 0.08 M), the aggregation suddenly formed, and the particle size became about 300 nm. However, with the further increase (from 0.1 to 0.3 M), the micelles instantaneously retrograded, showing a distribution form of monomers again (Figure 8b). These findings confirmed by DLS results were consistent with CD and fluorescence spectroscopy, showing that the particle size of buffalo β‐CN began to aggregate at pH 5.5 due to the intensive electrostatic repulsion. The changes in pH resulted in the formation of bigger particles by changing the ionization of the protein functional groups and the double‐layer thickness to affect protein‐protein interactions (Boulet et al., 2001). The surface charge of proteins neutralized by the increased NaCl concentration led to the reduction of intermolecular electrostatic repulsion and the exposure of hydrophobic groups. Consequently, internal proteins aggregated due to their strengthened hydrophobicity. When the NaCl concentration continued to increase, the protein electrostatic repulsion was raised, resulting in the dissociation of protein aggregation. Meanwhile, with modification of hydrophobic sites, free sulfhydryl was converted to disulfide bonds. Therefore, the hydrophilic‐hydrophobic balance affected its surface hydrophobicity and stability (Akintayo et al., 1999; Ouanezar et al., 2012). Nevertheless, changes in particle size with the increase in NaCl concentration were not clarified (Gualco, 2010). Mao et al. (2012) have figured out that the thiol‐disulfide interaction is enhanced with the increase in NaCl concentration. However, they have also reported that the higher the NaCl concentration, the smaller the particle size. Compared with the results reported by Ouanezar et al. (2012) on the CN micelle recombination in the acidification process using AFM, our study achieved an analog outcome with the method described in this paper.

## 4 CONCLUSIONS

In the present study, our data indicated that pH and NaCl concentration were critical factors in affecting structural features of buffalo β‐CN. Low pH did not seem to exert a great influence on the protein secondary structure at low protein concentrations. In contrast, it played an exceeding role in the protein secondary structure at a higher protein concentration and within a greater pH range, which was attributable to the increased intervention of hydrogen bond in the unordered structure. The high NaCl concentration and low pH caused the severe near‐complete loss of tertiary structure. In conclusion, the NaCl concentration, pH, and protein concentration played key roles in affecting the secondary, tertiary, and quaternary structures of buffalo β‐CN through altering electrostatic repulsion and hydrophilic‐hydrophobic balance. Therefore, our findings provided valuable insights into a better understanding of the mechanisms underlying the protein aggregation during the acidification of milk processing. Furthermore, the interactions between β‐CN and other proteins, as well as their functional properties, should be evaluated in future studies.

## CONFLICT OF INTEREST

All authors declare that there is no conflict of interest.

## Data Availability

Research data are not shared.
